# The Human Mouth, Its Care from Birth to the Completion of the First Dentition

**Published:** 1915-12

**Authors:** J. G. O’Neil


### THE HUMAN MOUTH, ITS CARE FROM BIRTH TO THE COMPLETION OF THE FIRST DENTITION.

J. G. O'NEIL, D.D.S., L.D.S.

This subject is confined definitely to the first two years of life, with advice on our part as to conditions, both normal and abnormal, with relation to the mouth, from a pathological and a physiological standpoint, covering, as it were, the very tenderest years, and the very time when that baby mouth requires the very best of care, and the most prudent advice within the area of dental treatment. We are conscious of the fact, yet how often we forget that there are in the human mouth today, as has been the condition through all the centuries, malignant factors of general infection and causes of disease wholly unperceived and neglected, and thus the oral cavity has ever been and still remains a prolific source of contagion. To conceive of the oral cavity as a vestibule of human life is a simile not inappropriate, for it is at this entrance that all the systemic stores, commissary supplies, fuels, etc., for nutrition are received and tested on their way to the stomach, the chief chemical laboratory and distributing center of the general system.

And not only this—it is here that the various foods, solids and liquids are incorporated with the oral secretions, are piped into the mouth from special glands, and the mass subjected to a process of macreation in preparation for deglutition. Engaged with these and other indispensable offices, the oral cavity is never wholly out of service, and literally it may be said it is never really cleansed. It is here that solid particles from the saliva, food remains, and other debris constantly deposit and accumulate, becoming cemented to the teeth, chiefly through inspissation of the viscid mucus perpetually oozing from many irregular glands concealed beneath the mucous membrane of the mouth, greatly augmenting the infection from this cavity. The air commonly diverted through it, especially in mouth breathers, and in sleep, becomes a purveyor of toxic emanations to the lungs, where it inevitably deposits its contagion in lung tissue or in the blood.

Necessarily the subject of such conditions—this vestibular cavity, with its twenty or thirty square inches of dentate surface, becomes quickly infested and infected with all manner of bacterial formations, decomposing food particles, stagnant inspissated septic matter from saliva, mucus and sputum, not infrequently with pus-exudition from irritated and inflamed gum margins; gaseous emanation from decaying teeth and putrescent pulp tissue; and the chemical toxins which result from the decomposition due to mingling of mouth secretions, maintained at the high normal of 98 degrees F.

The above is a picture of the conditions existing in a great many mouths of our present-day children, nor is it alone confined to the lower classes, but may be found to almost as great an extent in those children belonging to the higher classes.

We wonder in amazement at the accomplishments of man, his conquests of the air and water, in the sciences, in the arts, in building and architecture; in fact in every manner and form in which he has expended his efforts, both from an intellectual and a muscular standpoint, and if we only ponder a moment, we see as time advances that his efforts are those that are rapidly trending their way to that mighty goal of perfection. But is man in his own personal keeping in stride with the efforts which have been expended by him in all his enterprises?

And then—who can imagine, who dare predict, the social and economic revolution that must follow? Our social and business life today differs more widely from that of our grandfathers than theirs differed from the life of the Egyptians and the Babylonians, three thousand years ago.

What about our physical conditions? Are we as strong a race physically as were our ancestors?

Today the world is clamoring for perfection in man, as she is clamoring for perfection in all the products of his hands and brains. We want a healthy race of men, mentally, morally and physicially, and one of the means we have of accomplishing this end lies in the hands of the members of the dental profession, and that is our care of the human mouth, from the very beginning of life until we cease to tread this earthly sphere. There is a time in life when the attention which the human mouth should receive is the most vital one in all the decades, which we mortals pass through, and that is from birth to the completion of the temporary teeth. At this period the powers of immunity of the child are at their lowest ebb, and as the constructive process is at the highest point, the child requires more vitality in proportion to resistance powers than any other time in life.

At this time more than any other we desire that all the elements within the body of the child should work very harmoniously, and that nothing should exist that might impede progression of the child on to its goal of perfection.

Seventy-five years ago, but five per cent. of the population of the United States lived in the cities; today about seventy-five per cent. are so domiciled. Think of what that means to education! With ninety-five per cent. of the children out in the open fields, breathing the pure air of the open country, nourished with food that had not undergone adulteration, and trained in the various pursuits and crafts of the farmer boy and girl of that day, it is not surprising that little thought was given by the educator to the physical welfare of his pupil. When a child was close to nature, nature took care to correct the imperfections made by unintelligent civilization. But when outraged nature is overtaxed by the rush and hurry of modern society, she refuses to cope with the situation, and we are rearing a race of weaklings for future citizenship.

The great need of conserving the child for the nation should appeal strongly to us. We can not send him back to the country to live; we must deal with the condition as it exists, hence the training for a perfect life takes the place of his former simple life.

This training means advice, treatment, teaching, and all this work comes under the code that goes to make up the versatile profession of dentistry.

The mechanical engineer in charge of the building of skyscrapers in our large American cities insists on having the foundations as near perfection as his learned brain knows. He realizes that unless he is faultless in his details his super-structure will be a failure; so it is with life. We must begin with the very foundation, and see that this is secure, and that there are no hindrances which will retard that life in an absolutely normal physiological process.

We, as dentists, are really the guardians to the alimentary canal, and as one of the eminent physicians has said, that the majority of cases of some of our most dreaded diseases are contracted through the alimentary canal, then we must fortify our children by every means within our power, and the very time for us to do this is at the very commencement of life.

The oral cavity has such an influence over the rest of the body that all too often, due to abnormal conditions existing in the vestibular cavity, such ill effects result from these conditions that life is blighted and the physical, mental and moral conditions are so weakened that the effects from such a state only lead us to one conclusion, and that is, degeneracy.

The right of the child to be well born has its final sanction in the joy of living at all. For whatever the pessimist may say, life at its lowest and at its highest estate is the sum of all blessings whatsoever.

The eternal cosmic process would seem to have for its supreme goal the creation of life, and every creature born of this process shares the spirit that works through it all. It, too, has the will to live, and it, too, would create an even larger measure of life for itself and others. Thus it is that of all created things, whether sprung from the cosmic process or fashioned through the activities of living creatures themselves, the greatest is life. To live, and to have the endowments of life up to the limits one's nature imposes, is therefore an expression of the deepest purpose of the universe and of the soul of man. Therein is the fundamental right of every child born into the world.

If, therefore, life itself is the greatest of created things, and if the very purpose of the universe is fulfilled in such creation, what more noble work is there than that of the dental specialist, who by his work on the teeth and their contiguous parts aids materially in the betterment of the human race. All the creation of man's handicraft; all the creations of art, literature and science, all the creations of social philosophers and statesmen, are of secondary importance compared with the great work of a dentist in his fight for the betterment and perfection of the human race.

The mouth of the infant when first born is usually devoid of teeth. Cases are on record, however, in considerable numbers in which infants were born with one or several teeth. It has been stated that Richard Cœur de Lion, of England, and Louis XIV, of France, were born, each with several teeth. Haller, in his "Elements of

Physiology,'' mentions nineteen cases of children that were born with one or more teeth.

The normal activity of the salivary glands does not become established until the process of eruption of the teeth begins. Up to this time the diet of the infant has been composed only of milk, or ought to be, and there has been no occasion for the presence of saliva containing the special ferment, ptyalin. Upon the appearance of the teeth the child craves, and should have, a more solid diet, which its alimentary system is gradually being prepared to receive and digest. No more reprehensible practice can be indulged in by young mothers and nurses than that of feeding infants with materials that are so manifestly unfit, as starchy foods, fruits and meats, before the function of the salivary glands is established and a sufficient number of teeth have erupted to make mastication possible.

The eruption of the deciduous teeth is a physiologic process and in a normal child is productive of so little general or local disturbance that many times the teeth make their appearance within the mouth before the parent or the nurse has realized the fact that the process of ''teething'' has begun; while upon the other hand in children with impaired and low vitality it often plays an important part in exciting various morbid conditions of the digestive, nervous, respiratory and dermal systems. There is no doubt that parents are often unnecessarily anxious for their offspring during the period of primary eruption; yet it must be borne in mind that in certain temperaments, and under various physical conditions and environments, there is real danger present, and that morbid phenomena are sometimes excited which may progress to a fatal termination. When dentition is slow, and the gums swollen and painful, lancing may be necessary, or else applications of counter-irritants. Contemporaneously with the eruption of the teeth, there is a very important developmental process taking place in the follicular or glandular apparatus of the whole alimentary canal, in preparation for the necessary change soon to take place in the character of the food that the system will demand.

This is a physiologic process, and under normal conditions, when all the functions of the body are nicely balanced, progresses without the least disturbance of general health, but under opposite conditions it may be productive of serious gastric and intestinal complications, the causes of which, viz.; an unclean mouth, improper or spoiled food, etc., are often overlooked, and the disturbances which are the result of these indiscretions during the period that these important physiologic changes are taking place are attributed to morbid dentition.

The nervous system of the child at this period is also very impressible, the cerebro-spinal apparatus predominating to such an extent that slight irritations of almost any character in children of certain temperaments may be followed by more or less systemic disturbances, with elevation af temperature, vomiting, diarrhea, bronchitis and other catharrhal conditions, or reflex nervous phenomena like strabissmus, twitching of facial muscles, rolling of the eyes, convulsions or meningitis.

As soon as a child begins to take food its mouth should receive attention, but how few nurses or mothers ever think to cleanse a nursing baby's mouth. Much of the suffering, if not all of stomatitis, might be avoided by the nurse or mother in cleansing the mouth of the baby after feeding.

The mucous membrane of the mouth of the infant is very tender and sensitive, and often the seat of various superficial lesions, consequently in cleansing the mouth of the infant care must be exercised not to injure or in any way abrade the surface. The gums of the nursing infant are often studded with epithelial pearls which are in reality tiny retention cysts. The cysts may disappear by resorption or rupture spontaneously, and usually heal without marked symptoms. These minute lesions, however, may become infected from the presence of disease-producing micro-organisms growing in the mouth, resulting in ulcerated patches of more or less extent, which are sore and painful and frequently prevent the child from nursing, and in serious cases lead to grave forms of disease and sepsis.

The uncared for mouth of the child is never free from particles of coagulated and fermenting milk, which are the soil upon which many forms of harmful micro-organisms grow and flourish. These organisms may and do often attack the minute lesions above referred to and thus establish a follicular stomatitis. It stands to reason, therefore, that, if the cause can be excluded by keeping the mouth clean, the disease will be prevented. Nearly all the forms of stomatitis that affect infants and small children may be traced to an unclean condition of the mouth. This being the fact, no argument is needed to establish the relationship between cause and effect.

The best means of cleansing the mouth of the infant is done by wrapping a small piece of sterilized cotton fibre around the first finger of the right hand, that has been carefully washed with soap and sterilized water, and, after moistening the cotton in sterile water or saturated solution of boric acid, the finger is introduced and passed over the surface of the mouth and tongue, and particularly under the tongue and between the gums and cheeks, as these are the places where the particles of coagulated milk are usually found.

During the early stages of dentition the mouth should be inspected every day to see that there are no ulcerations of the gums at the points where the teeth are making their appearance. If the child cries when attempting to nurse, it is quite certain there is some form of stomatitis, and in cases of this kind it is well to consult with a physician.

As soon as the teeth begin to appear they should be kept scrupulously clean. This may be done at first by the method already described. After the teeth have fully erupted, the tooth brush will be indicated. This should be small in size, and the bristles soft. Camel's hair makes the best bristles for the baby's brush. In applying the brush special care should be exercised not to bruise or in any way injure the gums, or the mucous membrane of other portions of the mouth, as these tissues at this period are tender and easily abraded.

In case of infants the cleansing of the mouth twice each day will ordinarily be sufficient. This should be done morning and evening, preferably at the time of bath. After the teeth have erupted and the child is taking a mixed diet, the mouth and teeth should be cleansed three or four times each day, preferably on rising in the morning and after each meal. A thorough cleansing of the mouth before breakfast is more essential to good health than all other measures of personal hygiene combined.

Little children should be taught to use the tooth brush as soon as the imitative faculties begin to develop. This may be done by the nurse or mother brushing her own teeth before the child, who has been furnished with an appropriate brush. The lessons, for it will take many, should be carefully and slowly taught, so that the child will comprehend all the movements that are required to reach all parts of the mouth and teeth.

The child should be taught to view the teeth in a looking-glass to see if they are clean, and compared with those of the mother or nurse. In this way the pride of the child is stimulated and rivalry awakened to have the nicest looking teeth.

Of course it is not expected that children of three years of age can be taught to care for their own teeth; this must be the duty of the nurse or mother. A beginning, however, can be made at this period, and a habit formed as the months and years go by that will cling to the child through all after life. The importance of this early training can not be overestimated, as it insures mouth comfort, and good health and long life, barring accidents.

The individual who has been brought up from early childhood to give proper care to the cleanliness of the mouth and teeth, is rendered quite miserable if for any reason he is obliged to neglect even for a single day this brushing of the teeth.

At no period in the life of the child is oral hygiene or mouth cleanliness of such importance as during illness. At such time the secretions of the mouth are more or less changed, and frequently are decidedly acid in reaction; while the micro-organisms of the mouth are less disturbed than during health, and as a consequence grow and propagate rapidly. Great damage is often done to the teeth during illness from neglect to keep the mouth clean. Of course it should be understood that during a severe illness it may not be possible to carry out a careful and thorough regime of mouth cleaning on account of the disturbances to the child. A little careful cleansing of the mouth and teeth will not be harmful to the general condition of the child at such times, while in a majority of instances it will prove restful and refreshing. The means already described for cleansing the mouths of infants can be employed in these cases with ease.

If mucous deposits of dark color form upon the teeth, they can easily be removed with a chisel-shaped piece of orangewood, and charged with a little precipitated chalk, and the teeth wiped off with a little cotton moistened with a saturated solution of boric acid.

In conclusion I shall quote what Dr. Osler said in a recent address before a gathering of dentists: "You have just one gospel to preach, and you have to preach it early and you have to preach it late, in season and out of season. It is the gospel of cleanliness of the mouth, cleanliness of the teeth, cleanliness of the throat; these three things must be your text through life.''—*Dominion Dental Journal.*

